# Vascular Effects of Advanced Glycation End-Products: Content of Immunohistochemically Detected AGEs in Radial Artery Samples as a Predictor for Arterial Calcification and Cardiovascular Risk in Asymptomatic Patients with Chronic Kidney Disease

**DOI:** 10.1155/2015/153978

**Published:** 2015-03-17

**Authors:** Katarzyna Janda, Marcin Krzanowski, Mariusz Gajda, Paulina Dumnicka, Ewa Jasek, Danuta Fedak, Agata Pietrzycka, Marek Kuźniewski, Jan A. Litwin, Władysław Sułowicz

**Affiliations:** ^1^Chair and Department of Nephrology, Jagiellonian University Medical College, Cracow, Poland; ^2^Chair and Department of Histology, Jagiellonian University Medical College, Cracow, Poland; ^3^Department of Medical Diagnostics, Jagiellonian University Medical College, Cracow, Poland; ^4^Chair of Clinical Biochemistry, Jagiellonian University Medical College, Cracow, Poland; ^5^Pharmacobiology Department of Pharmacy, Jagiellonian University Medical College, Cracow, Poland

## Abstract

*Objectives.* Our aim was to determine whether vascular deposition of advanced glycation end-products (AGEs) is associated with arterial calcification and cardiovascular mortality in chronic kidney disease (CKD) patients and to assess the relationships between vascular content of AGEs and selected clinical and biochemical parameters.* Materials and Methods.* The study comprised 54 CKD patients (33 hemodialyzed, 21 predialyzed). Examined parameters included BMI, incidence of diabetes, plasma fasting glucose, AGEs, soluble receptor for AGEs and 2,2-diphenyl-1-picrylhydrazyl (DPPH) scavenging, serum C-reactive protein (hsCRP), plasminogen activator inhibitor-1 (PAI-1), and fetuin-A. Fragments of radial artery obtained during creation of hemodialysis access were stained for calcifications using alizarin red. AGEs deposits were identified immunohistochemically and their relative content was quantified.* Results.* Vascular content of AGEs was positively correlated with BMI, hsCRP, fetuin-A, PAI-1, and DPPH scavenging in simple regression; only fetuin-A was an independent predictor in multiple regression. There was a significant positive trend in the intensity of AGEs immunostaining among patients with grades 1, 2, and 3 calcifications. AGEs immunostaining intensity predicted 3-year cardiovascular mortality irrespective of patient's age. *Conclusions.* The present study demonstrates an involvement of AGEs in the development of medial arterial calcification and the impact of arterial AGE deposition on cardiovascular mortality in CKD patients.

## 1. Introduction

Advanced glycation end-products (AGEs) and their receptor play an important role in the pathogenesis of vascular damage and cardiovascular disorders, especially in patients with diabetes and chronic kidney disease (CKD). AGEs accumulate in various organs of the body including the heart and large blood vessels, leading to accelerated plaque formation as well as increased cardiac fibrosis with consequent effects on cardiac dysfunction. Medial arterial calcification (MAC) has been shown to increase the accumulation of AGEs on elastin and collagen in the aorta of patients and in femoral arteries in animal model [1]. Elastin stiffening induced by AGE cross-links appeared in parallel with an increase in calcium uptake by elastin. The calcium binding was also increased in collagen exposed to glucose. It was found that diabetic serum induced a receptor for advanced glycation end-products- (RAGE-) dependent calcification of vascular smooth muscle cells (VSMCs). AGEs not only enhance the affinity of the extracellular matrix components for calcium but also induce signaling pathways, resulting in cell phenotype changes. RAGE activation caused the osteogenic differentiation of VSMC by the activation of the extracellular-signal-regulated kinases 1/2 (ERK1/2) pathways [2].

The initial accumulation of AGEs in the vascular tissue could exacerbate local inflammation and lead to downstream cellular activities that favor cell-mediated vascular calcification by activation of RAGE [3, 4]. Ren et al. [3] demonstrated that, in response to AGEs, rat aortic VSMCs differentiate into cells that exhibit an osteoblast-like phenotype, characterized by the deposition of calcium in the extracellular matrix and the expression of genes normally restricted to mineralized tissues, such as osteopontin and alkaline phosphatase. RAGE has emerged as a central regulator of vascular inflammation and atherosclerosis. Soluble RAGE (sRAGE) has an anti-inflammatory effect by quenching ligands for RAGE. The circulating sRAGE level was inversely associated with vascular calcification scores in hemodialyzed patients irrespective of the severity of systemic inflammation [5].

The aim of the present study was to determine whether AGE content in the arterial wall, as expressed by the intensity of immunostaining, is significantly associated with histologically assessed arterial calcification and the incidence of cardiovascular events in patients with stage 5 chronic kidney disease. Moreover, we examined possible correlations of vascular AGEs deposits and calcifications with the levels of circulating AGEs and sRAGE as well as markers of bone and mineral metabolism, inflammation, and oxidative stress.

## 2. Materials and Methods

### 2.1. Patients

The study population consisted of 54 patients (stage 5 of CKD), including 33 on maintenance hemodialysis (HD) and 21 on predialysis. The study included patients who underwent the first creation of arteriovenous fistula for hemodialysis access. The majority of the subjects were men (34, i.e., 63%). The mean age at the beginning of the study was 61 ± 16 years. The mean arterial pressure (MAP) was calculated according to the formula: MAP = (SBP + 2 × DBP)/3, where SBP = systolic blood pressure and DBP = diastolic blood pressure.

The data on mortality was collected over a period of three years. All deaths occurred in hospital and causes of death were determined using history documentation.

The study was approved by the Bioethics Committee of the Jagiellonian University and all patients signed an informed consent for their participation.

### 2.2. Laboratory Tests

Blood samples of the patients were obtained at the beginning of the study in the morning before creation of arteriovenous fistula for hemodialysis access. Serum samples for ELISA tests were aliquoted and stored at −70°C until being assayed (no longer than 3 months). Plasma samples used to assess oxidative stress parameters were protected from light, placed on ice and centrifuged within 2 hours after collection, then aliquoted, and stored at −30°C until analysis (no longer than one month). In all patients, the following biochemical parameters were assessed: serum concentrations of total cholesterol, HDL-cholesterol, LDL-cholesterol, triglycerides (TG), serum creatinine, albumin, glucose, parathyroid hormone (iPTH), total calcium (Ca) and phosphate (Pi), advanced glycation products (AGEs), the soluble form of receptor for advanced glycation end-products (sRAGE), inflammatory markers, high sensitive C-reactive protein (hsCRP), interleukin-6 (IL-6), plasminogen activator inhibitor PAI-1 (PAI-1), and fetuin-A, and calcification markers, osteopontin (OPN), osteoprotegerin (OPG), osteocalcin (OC), and fibroblast growth factor 23 (FGF-23).* Homeostasis Model of Assessment*-*Insulin Resistance* (HOMA-IR) was calculated by application of the international formula: fasting insulin (*μ*IU/mL) × fasting glucose (mmol/L)/22.5. The estimated glomerular filtration rate (eGFR) was calculated by* Modification of Diet in Renal Disease *(MDRD) formula: eGFR = 186 × [serum creatinine (*μ*mol/L) × 0.0113]^−1.154^  × age^−0.203^  × (0.742 for women).

Routine biochemical tests were carried out using automatic biochemical analyzers: Hitachi 917 (Hitachi, Japan) and Modular P (Roche Diagnostics, Mannheim, Germany). Concentrations of hsCRP were measured using immunonephelometric method and Nephelometer BN II (Siemens Healthcare Diagnostics, Germany).

Inflammatory and calcification markers were determined using ELISA microplate immunoassays and ELX808 automatic reader (BioTEK Instruments Inc., Vermont, USA). The following sets of kit reagents were applied: IL-6 (R&D Systems, Minneapolis, MN, USA), PAI-1 (Human Serpin/PAI-1, R&D Systems, Minneapolis, MN, USA), fetuin-A (BioVendor, Czech Republic), OPN (R&D Systems, Minneapolis, MN, USA), OC (METRA, Germany), OPG (QUIDEL, BioVendor, Czech Republic), and FGF-23 (C-Term) (Immutopics Inc., San Clemente, USA).

AGE and sRAGE concentrations were measured with commercially available Human ELISA kits (BIOMATIK, Life Science Products and Services, Canada) according to the manufacturer's protocol. Spectrophotometric measurements were performed using microplate reader Polar Star Omega (BMG Labtech, Germany). Total antioxidant capacity of plasma was assessed as the ability of plasma to reduce Fe^3+^ to Fe^2+^ (ferric reducing ability of plasma—FRAP), according to Benzie's method [6]. Radical scavenging capacity of plasma was estimated by DPPH radical scavenging assay as described elsewhere. Ferric reducing ability of ascorbate in plasma (FRASC) was measured spectrophotometrically [6, 7].

### 2.3. Histology

Small fragments of radial artery wall, approximately 5 × 2 mm in size, were collected during the first creation of arteriovenous fistula for hemodialysis access. The samples were fixed overnight in 10% phosphate-buffered formalin and then rinsed in PBS and soaked in 30% sucrose. The material was snap-frozen and tissue blocks were positioned in a cryostat to allow cutting sections in a longitudinal plane of the vessel encompassing the entire thickness of the vascular wall. Serial 10 *μ*m thick cryosections were cut and thaw-mounted on poly-L-lysine coated slides. Sections were stained routinely with Mayer's haematoxylin and eosin (HE) for general morphology and with alizarin red for calcifications. AGE deposits were detected using indirect immunofluorescence labelling. Briefly, after preincubation with 5% normal goat serum for 20 min the sections were incubated overnight with polyclonal rabbit anti-AGE antibody (ab23722, Abcam, Cambridge, UK; 1 : 200). After rinsing in PBS sections were incubated with secondary Cy3-conjugated goat anti-rabbit serum (111-165-164, Jackson IR, West Grove, PA; 1 : 400) for 1 h and then rinsed and mounted in glycerin/PBS. To verify specificity of the immunostaining control reactions omitting primary antibody and/or secondary antiserum were applied.

Sections were examined under Olympus BX-50 microscope (Olympus, Tokyo, Japan) in brightfield mode for histological staining and in fluorescence mode for immunolabeling. Images were acquired using Olympus DP-71 digital CCD camera controlled by Olympus AnalySIS FIVE software. All images from immunolabeled sections were recorded under standardized conditions of the fluorescent lamp (identical power of the excitation light) and of the camera (sensitivity and acquisition time). The advancement of vascular calcification was semiquantitatively assessed in alizarin red-stained sections by two independent observers. The degree of mineralization was classified according to the following scale: 0, no mineral content; 1, a few small dispersed concretions; 2, numerous small dispersed concretions; 3, larger granular concretions; and 4, large areas occupied by fused mineral deposits. AGE deposition was quantified in the arterial media by measuring mean red fluorescence intensity (range 0–255, arbitrary units) in the analyzed area and expressed as the mean fluorescence intensity for each vessel. From each sample three sections from different depths of the tissue block were analyzed. The appropriate algorithms for quantification were adopted in AnalySIS FIVE software.

The reproducibility of the morphological analysis was confirmed by Bland-Altman method and by calculating intraclass correlation coefficient (ICC) which was 0.88.

### 2.4. Statistical Methods

The data are expressed as the number of patients (percentage of the particular group) for categories and as mean ± SD or median (lower-upper quartile) for continuous variables, according to the distribution. Shapiro-Wilk test was used to check for normality. Contingency tables were analyzed with Pearson chi-squared test. Student's *t*-test or Mann-Whitney test was used for simple comparisons between the groups. Simple and multiple linear regressions were calculated after log-transformation of right-skewed variables. Multiple regression included the predictors significantly correlated with dependent variable in simple regression (*P* < 0.05). Variance inflation factors (VIF) were calculated for the predictor variables; VIF above 5 were not accepted. The association between the intensity of immunostaining for AGEs and the degree of vascular calcification was tested with one-way ANOVA and trend analysis. Logistic regression analysis was used to study the associations with mortality; odds ratio (OR) for 1 SD increment was reported with 95% confidence interval (95% CI). All tests were two-tailed and the results were considered significant at *P* ≤ 0.05. The computations were performed using Statistica 10 software (StatSoft, Tulsa, OK, USA).

## 3. Results

### 3.1. Characteristics of the Study Group

Clinical characteristics of the patients and the results of the laboratory tests are presented in Table 1.

According to the results of AGE immunostaining measurements, the patients were divided into two groups: those showing the vascular AGE content above and below the median value (48.3). Patients with high content of AGEs in radial artery had higher concentrations of fetuin-A and PAI-1 as well as higher free radical scavenging capacity of plasma (as shown by DPPH scavenging assay).

The AGE content in radial artery did not differ between the diabetics and nondiabetics (54.9 (42.2–85.9) versus 45.9 (30.5–72.1); *P* = 0.2) and did not depend on hemodialysis status (HD versus predialysis: 57.0 (33.0–81.8) versus 42.8 (34.6–60.9); *P* = 0.3). Similarly, serum concentrations of AGEs and sRAGE as well as the ratio of AGEs/sRAGE did not differ significantly between patients with and without diabetes or between dialyzed and predialyzed subjects.

### 3.2. Histological Findings

Calcifications of various grades were detected in radial artery samples of 30 patients (56%): 11 patients had grade 1, 5 grade 2, 8 grade 3, and 6 grade 4 calcifications.

**Figure 1:**
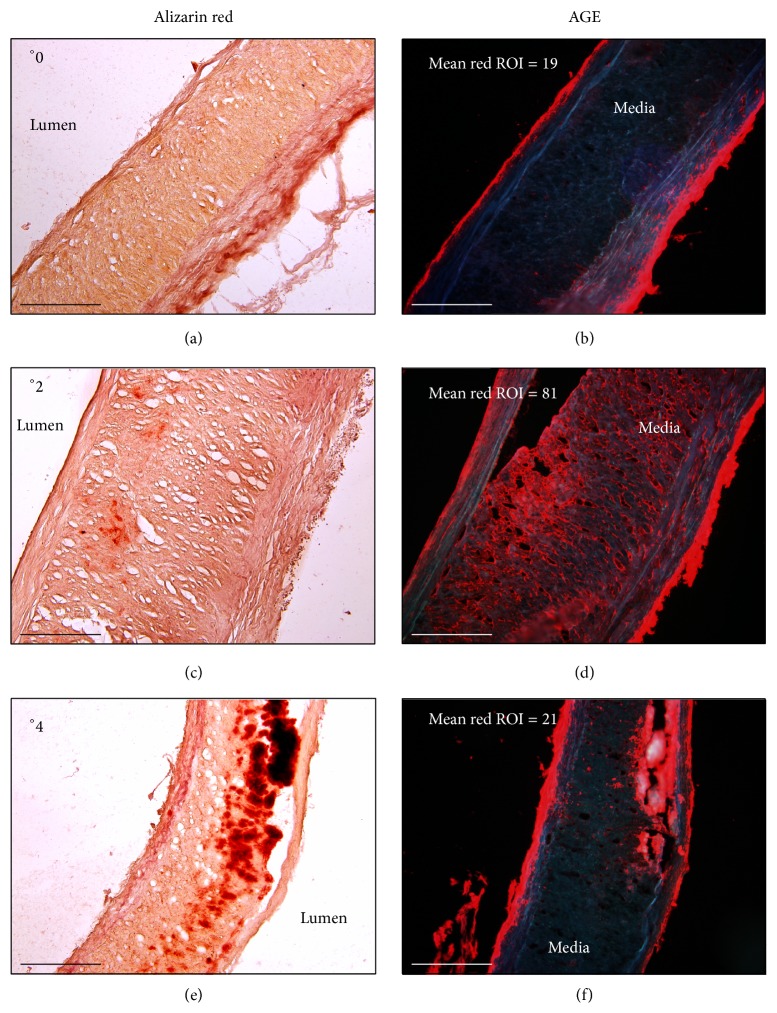
Consecutive sections of radial arteries stained with alizarin red (a, c, and e) and immunostained for AGE (b, d, and e) showing calcifications of various grades 0 (a), 2 (c), and 4 (e). AGE deposits immunolabeled in red color presenting low (b and f) and high (d) intensities of the immunofluorescence in media. Bar = 200 *μ*m.

AGE immunohistochemistry showed intense reaction in the intima and in the adventitia of the examined vessels. In the media, AGE deposits were seen in various grades of advancement and they were mostly localized extracellularly between smooth muscle cells (Figures 1(c) and 1(d)). Since the immunostaining in intima and adventitia may result from nonspecific binding of AGE antibodies to glycoproteins (e.g., collagen), only AGEs located in the media were considered for further measurements and comparisons.

### 3.3. Correlations of AGE Content in Radial Artery with Clinical and Biochemical Parameters

The intensity of AGEs staining in radial arteries of stage 5 CKD patients positively correlated with BMI, hsCRP, fetuin-A, PAI-1 concentrations, and DPPH scavenging (Table 2; right-skewed variables were log-transformed). Neither AGEs, sRAGE, nor AGEs/sRAGE ratio correlated with the intensity of radial artery staining for AGEs. In multiple regression, only fetuin-A predicted the intensity of AGEs staining in radial artery wall independently of log (BMI), log (hsCRP), log (PAI-1), and log (DPPH scavenging) (Table 2). Variance inflation factors (VIF) for the predictor variables were <2.

### 3.4. Association of AGE Content in Radial Artery and AGEs, sRAGE, and AGEs/sRAGE in Serum with Vascular Calcification

**Figure 2:**
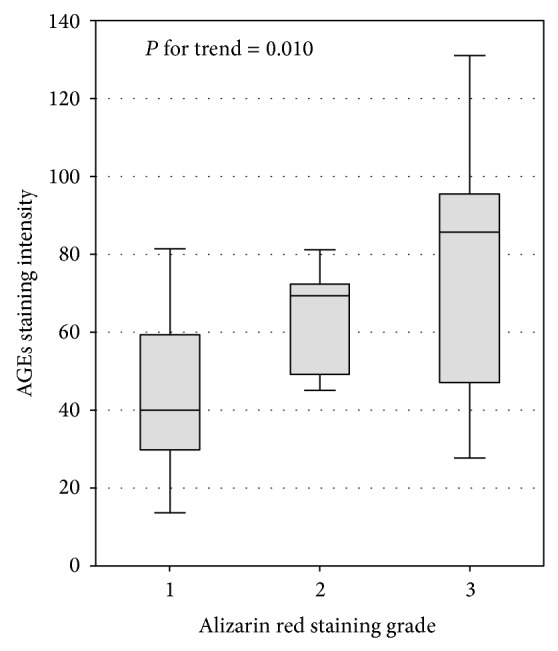
The association between alizarin red staining and AGE immunostaining intensity in radial arteries of patients with nonmassive vascular calcifications.

We did not observe significant differences regarding AGE and sRAGE serum concentrations, AGEs/sRAGE ratio, or the intensity of vascular staining for AGEs between patients with and without vascular calcifications. However, there was a significant positive trend in the intensity of AGE staining among patients with alizarin red staining grades 1, 2, and 3 (one-way ANOVA: *F*_2,21_ = 4.13; *P* = 0.031; *P* for trend = 0.010; Figure 2). This trend was also significant after excluding diabetic patients from the analysis (*P* = 0.001).

### 3.5. Association of AGE Content in Radial Artery with Mortality

**Figure 3:**
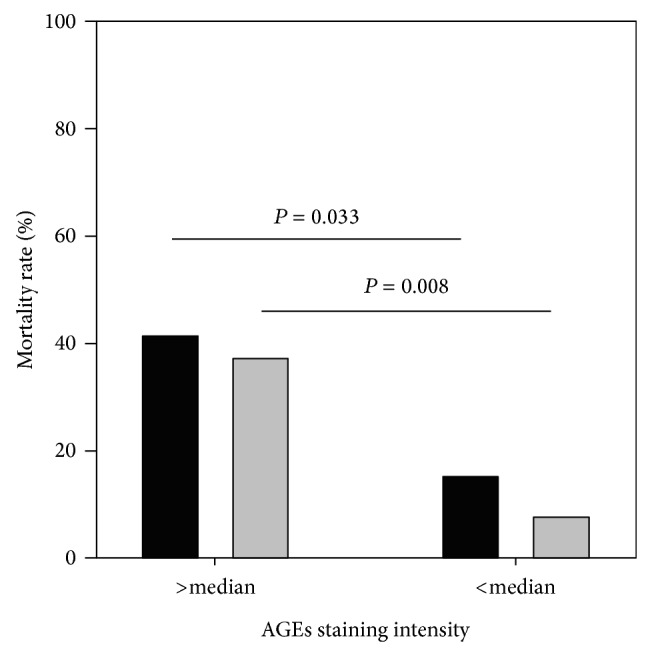
All-cause (black bars) and cardiovascular (grey bars) mortality rate in patients with high and low AGE content in radial artery.

During 3-year observation period, 15 (28%) patients died, including 12 due to cardiovascular causes (myocardial infarction in 5 cases, heart failure in 6, and cerebral stroke in 1 patient). More deaths (11 versus 4; *P* = 0.033), including cardiovascular ones (10 versus 2; *P* = 0.008), occurred in patients with high (i.e., above median) intensity of vascular staining for AGEs (Figure 3). In logistic regression analysis, the intensity of AGE staining significantly predicted cardiovascular (not all-cause) mortality (OR 2.10 (1.04–4.26) per 1 SD increment; *P* = 0.030), irrespective of patient's age. There were no associations of plasma AGE and sRAGE concentrations or AGEs/sRAGE ratio with overall and cardiovascular mortality.

## 4. Discussion

To the best of our knowledge, this is the first report where AGE content was measured using quantitative immunochemistry in human tissues obtained intravitally. Using small samples of radial artery obtained during the creation of arteriovenous fistula for hemodialysis access, we showed for the first time the association between the intensity of AGE staining and the severity of medial arterial calcification (MAC) in patients with renal failure. Earlier studies examining the impact of AGEs on the calcification processes were carried out primarily in diabetic patients or using animal experimental models in vivo and in vitro [1, 8–14]. Furthermore, earlier studies mainly focused on the associations of AGEs with atherosclerosis in patients without CKD. Baumann et al. [15] demonstrated that the advanced glycation end-product N^*ε*^-carboxymethyllysine (CML) was present in the subendothelial space, especially in the atheromatous lesions of human carotid artery obtained during carotidectomy. In contrast, our study demonstrated the presence of AGEs deposits in the medial layer of human radial arteries. This is the first study showing the presence of AGEs in this layer of artery walls in living patients. Similarly, large and medium-sized mineral deposits were found most frequently in the vascular media.

MAC represents calcification that proceeds via matrix vesicle-nucleated mineralization accompanied by apatitic calcium phosphate deposits in the arterial media in the absence of atheroma. Osteoblast-like cells differentiate in the vessel wall from vascular smooth muscle cells and multipotent vascular mesenchymal progenitors. These cells, as well as the recruitment of undifferentiated progenitors of the osteochondrocyte lineage, play a critical role in the calcification process [16]. Transcription factors expressed by these cells such as Msx 2, Osterix, and RUNX2 are crucial in the programming of osteogenesis [2, 9]. However, most studies assessing the risk of calcification use noninvasive methods, such as CT scan and pulse wave velocity, without specifying the nature of the changes in the vessel (i.e., atherosclerotic changes or MAC). In particular, the data on the relationship between AGEs, sRAGE levels, and calcification were collected on the basis of such studies [17, 18]. Multiple factors contribute to the induction and progression of MAC, including inflammation, oxidative stress, bone, and mineral disorders, as well as AGEs in diabetic patients [8, 15, 19]. We observed the association between AGE content in the medial layer of radial artery with the degree of MAC in CKD patients. Importantly, the association was significant after excluding subjects without diabetes. Also, the intensity of staining for AGEs did not differ between diabetics and nondiabetics.

Interestingly, we found no significant relationship between plasma AGEs and sRAGE concentrations and the content of AGEs in the media of radial arteries. We have not found any studies on the relationship between plasma AGEs and the magnitude of AGEs deposits in arterial wall. According to our results, accumulation of AGEs in the vessel wall does not directly correlate with its concentration in plasma. However, we used a nonselective ELISA method detecting various types of AGEs; thus we cannot exclude that only selected AGE types form arterial wall deposits.

BMI and parameters of inflammation and oxidative stress are recognized as the factors associated with vascular calcification [20, 21]. Our results showed a relationship between hsCRP and the severity of AGE deposition in the medial layer of the radial artery. The initial accumulation of AGEs in vascular tissue could exacerbate local inflammation and lead to downstream cellular events that favour cell-mediated vascular calcification via activation of RAGE. On the other hand, chronic inflammation may increase the calcification processes in renal failure [22, 23]. Vascular reactive oxygen species (ROS) contribute to vascular functional and structural alterations. High levels of ROS can result in the reduction of proliferation, increased apoptosis, and modulation of differentiation. AGEs are able to quench nitric oxide (NO) and increase the generation of ROS such as peroxynitrite [10]. Our study showed a relationship between DPPH scavenging and the content of AGEs in the vessel wall. Studies using animal models confirm this suggestion. In rat model of diabetes that shared similarities with human type 2 diabetes, calcification was induced by vitamin D3 and nicotine. The authors showed a significant increase in aortic calcium content, levels of aorta AGEs, malondialdehyde content, ALP protein levels, and RAGE expression together with significant decrease in Cu/Zn superoxide dismutase (SOD) activity [10]. SOD acts as the first line of defense against oxygen free radical mediated damage by catalyzing the dismutation of superoxide anions. Brodeur et al. [1] demonstrated that AGE inhibitors prevent time-dependent accumulation of AGEs in femoral arteries in diabetic rats. This effect was accompanied by a reduction in diabetes-accelerated calcification. Ex vivo experiments showed N-methylpyridinium as an agonist of RAGE-mediated calcification of diabetic femoral arteries, a process inhibited by antioxidants and various inhibitors of signaling pathways associated with RAGE activation. The importance of oxidative stress was demonstrated by the reduction of femoral medial artery calcification in diabetic rats treated with apocynin, an inhibitor of reactive oxygen species production. Engagement of RAGE with AGEs elicits intracellular ROS generation and subsequently activates mitogen-activated protein kinase and nuclear factor kappa-B signaling, followed by production of several inflammatory species and profibrotic factors such as vascular cell adhesion molecule-1 (VCAM-1), intracellular adhesion molecule-1 (ICAM-1), plasminogen activator inhibitor-1 (PAI-1), and monocyte chemoattractant protein-1 (MCP-1), involved in the progression of atherosclerosis [12]. We have shown a relationship between the plasma levels of PAI-1 and the intensity of immunostaining for AGEs in the media of radial artery walls. However, the role of PAI-1 in the development of MAC has not been elucidated.

Fetuin-A is a negative acute-phase protein, which acts as a potent calcification inhibitor and an antagonist of TGF-*β*. Thus, fetuin-A serum concentrations are influenced by chronic inflammation and actively affect fibrosis and calcification processes. Several studies have shown that fetuin-A is a potent inhibitor of calcifications both in vivo and in vitro [24, 25]. In our study, fetuin-A was an independent predictor for deposits of AGEs in radial artery walls. Roos et al. [26] examined the correlation between FGF-23, fetuin-A, and coronary artery calcium score in patients with normal renal function. They found no correlation between the presence of noncalcified plaques and coronary artery stenosis and serum fetuin-A concentrations. However, correlations between low fetuin-A levels and vascular or valvular calcifications in CKD patients have been shown in several studies [27, 28]. Schlieper et al. [29] performed ultrastructural analysis of iliac artery segments of dialysis patients and observed an association between microcalcifications in the media and local content of calcification inhibitors including fetuin-A. Similarly, Maréchal et al. [30] demonstrated that low serum fetuin-A concentrations were independently associated with aortic calcifications and higher risk of CV events and mortality. Our study showed a positive relationship between serum level of fetuin-A and the intensity of staining for AGEs in arterial media. Moreover, fetuin-A was the only independent predictor of AGE content in the arterial media and since that content was correlated with degree of calcification at early stages of MAC development, increase in fetuin-A serum level seems to be associated with the initial progression of arterial calcification. This result seems to contradict the anticalcific activity of fetuin-A; however it can suggest a possible protective upregulation of fetuin-A at the early stages of exposure to the procalcific and proinflammatory uremic environment.

In patients suffering from CKD, medial calcification of peripheral arteries is a strong independent predictor of cardiovascular morbidity and mortality [31–34]. Our study showed for the first time that the intensity of AGE deposition in arterial media significantly predicted not only the advancement of medial arterial calcification but also cardiovascular mortality in CKD patients. In the literature only a few reports point to the relationship between the concentration of serum AGEs and cardiovascular risk but there are no data on the relationship between AGE deposits in blood vessels and mortality [35, 36].

## 5. Conclusions

The present study demonstrates an involvement of AGEs in the development of medial arterial calcification and the impact of arterial AGE deposition on cardiovascular mortality in CKD patients.

## Figures and Tables

**Table 1:** Clinical and laboratory characteristics of stage 5 CKD patients at the start of the study.

	Intensity of AGEs staining > median (*N* = 27)	Intensity of AGEs staining ≤ median (*N* = 27)	*P*
Age, years	63.1 ± 14.9	59.2 ± 17.2	0.4
Male gender, *N* (%)	18 (67)	16 (59)	0.6
Hemodialyzed, *N* (%)	19 (70)	14 (52)	0.2
Dialysis duration, months^a^	7 (2–32)	11 (2–38)	0.7
BMI, kg/m^2^	27.5 ± 6.1	25.1 ± 5.4	0.067
Diabetes, *N* (%)	9 (33)	7 (26)	0.6
Active smoking, *N* (%)	10 (37)	6 (22)	0.2
Hypertension, *N* (%)	11 (41)	11 (41)	1.0
MAP, mmHg	102 ± 12	103 ± 13	0.6
Serum creatinine, *μ*mol/L	464 (405–596)	446 (313–510)	0.1
eGFR (MDRD), mL/min/1.73 m^2a^	12 (10–14)	14 (10–19)	0.4
Albumin, g/L	41.7 ± 3.4	40.1 ± 6.0	0.4
Total cholesterol, mmol/L	4.65 ± 1.27	4.97 ± 1.76	0.6
HDL-cholesterol, mmol/L	1.23 ± 0.38	1.30 ± 0.40	0.6
LDL-cholesterol, mmol/L	2.56 ± 1.00	2.70 ± 1.41	0.8
Triglycerides, mmol/L	1.98 ± 1.17	2.10 ± 1.00	0.4
Fasting glucose, mmol/L	5.30 (4.80–7.90)	5.00 (4.40–5.40)	0.2
Insulin, *μ*U/mL^b^	10.7 (6.2–23.8)	8.4 (5.2–12.1)	0.3
HOMA-IR^b^	2.28 (1.32–4.85)	1.68 (1.08–2.68)	0.2
Ca × Pi, mmol^2^/L^2^	3.59 (2.87–4.46)	3.04 (2.91–3.70)	0.4
iPTH, pg/mL	286 (186–524)	265 (153–343)	0.4
OPN, ng/mL	340 (225–681)	315 (215–438)	0.3
OPG, pmol/L	7.80 (5.76–10.61)	7.43 (3.03–12.52)	0.5
OC, ng/mL	44.2 (33.6–78.2)	41.8 (24.6–61.8)	0.2
FGF-23, RU/mL	1148 (487–5066)	970 (435–1468)	0.3
Fetuin-A, g/L	0.27 ± 0.06	0.23 ± 0.04	0.042
CRP, mg/L	9.64 (2.97–19.00)	4.80 (2.19–17.90)	0.5
IL-6, pg/mL	4.58 (1.86–6.36)	3.89 (2.18–8.22)	0.7
uric acid,	349 (234–426)	355 (259–432)	0.9
FRAP, mM/L	0.75 (0.62–1.21)	0.74 (0.52–1.06)	0.5
FRASC, *μ*M/L	46.8 (41.0–58.9)	50.2 (43.1–57.3)	0.9
DPPH, %	43.4 (37.4–68.0)	36.8 (33.0–40.5)	0.007
PAI-1, ng/mL	1.92 (1.58–2.57)	1.16 (0.95–1.77)	0.011
Plasma AGEs, ng/mL	537 (274–741)	492 (251–2570)	0.8
Plasma sRAGE, ng/mL	1.75 (1.09–2.73)	1.91 (1.32–2.72)	0.7
Plasma AGEs/sRAGE	412 (116–777)	264 (132–1455)	0.7
AGEs in radial artery, arbitrary units	80.9 ± 18.7	33.6 ± 9.5	—

^a^Data for hemodialyzed patients; ^b^data for patients without diabetes.

**Table 2:** Predictors of AGE staining intensity in radial arteries in simple and multiple linear regressions.

	Simple regression		Multiple regression	
	*R*	*P*	Beta ± SE	*P*
log(BMI)	0.27	0.047	0.16 ± 0.15	0.3
log(hsCRP)	0.29	0.045	0.22 ± 0.14	0.1
Fetuin-A	0.46	0.002	0.32 ± 0.15	0.045
log(PAI-1)	0.40	0.005	0.07 ± 0.16	0.7
log(DPPH scavenging)	0.36	0.014	0.22 ± 0.15	0.2
Whole model	—		*R* ^2^ = 0.32; *P* = 0.013	

*R*: correlation coefficient, SE: standard error, and *R* ^2^: coefficient of determination.
